# Association of the cyclooxygenase-2 1759A allele with migraine in Chinese Han individuals

**DOI:** 10.1371/journal.pone.0239856

**Published:** 2020-09-30

**Authors:** Xinying Guan, Changhong Dong, Pinhuan Zhu, Cheng Chen, Teng Wang, Mengping Wu, Xin Dong

**Affiliations:** 1 Department of Neurology, The Affiliated Hospital of Kangda College of Nanjing Medical University/The First People’s Hospital of Lianyungang, Lianyungang, Jiangsu Province, China; 2 Department of Clinical Medicine, Xuzhou Medical University, Xuzhou, Jiangsu Province, China; 3 Department of Neurology, The First Affiliated Hospital of Nanjing Medical University, Nanjing, Jiangsu Province, China; 4 Department of Statistics, The Affiliated Hospital of Kangda College of Nanjing Medical University/The First People’s Hospital of Lianyungang, Lianyungang, Jiangsu Province, China; National Institutes of Health, UNITED STATES

## Abstract

Cyclooxygenase-2 (COX-2) is known to be involved in the pathogenesis of migraine, and some polymorphisms are known to affect the expression of COX-2. This retrospective case-control study aimed to explore the associations between the -765 G>C (rs20417), -1759 G>A (rs3218625), and -8473 C>T (rs5275) COX-2 polymorphisms and migraine in Chinese Han individuals. One hundred and ten unrelated Han Chinese patients with migraine and 108 healthy controls were recruited between 03/2014 and 08/2016 at the First Affiliated Hospital of Nanjing Medical University and the First People's Hospital of Lianyungang City. The genotypes of all polymorphisms in controls followed the Hardy-Weinberg equilibrium (P = 0.215, P = 0.884, and P = 0.689). There were differences in the genotype and allele distributions of the COX-2-1759G>A (Gly587Arg) polymorphism between the migraine and control groups (P = 0.038 and P = 0.040, respectively). Compared with the COX-2-1759AG genotype, GG genotype carriers had an increased risk of migraine (odds ratio (OR) = 8.720, 95% confidence interval (CI): 1.072–70.960, P = 0.038). The frequency of the COX-2-1759A allele in patients with migraine was significantly lower than the controls (OR = 0.119, 95%CI: 0.015–0.957, P = 0.040). Adjusted age and sex, a statistical difference was found in the dominant model of COX-2-1759 G>A (OR = 0.118, 95% CI 0.014 to 0.962, P = 0.046). No significant difference was detected regarding the -765G>C and -8473T>C polymorphisms between the two groups. The COX-2 1759A allele might be involved in the development of migraine in Chinese Han individuals, but this will have to be confirmed in large-scale studies.

## Introduction

Migraine is a common primary headache disorder of unclear etiology. It is recurrent in nature and classically presents as moderate-to-severe head pain lasting 4–72 hours; it is typically unilateral with a pulsating quality, accompanied by nausea, vomiting, photophobia, and/or phonophobia, and may be preceded by an aura that consists of sensory, motor, or language symptoms [1–3]. The cumulative lifetime incidence is 43% in women and 18% in men [4]. The prevalence is about 11.8% in the world, 15% in the USA, and 9.3% in mainland China [5, 6]. Migraine is classified into two primary subtypes: migraine with aura (MA) and migraine without aura (MO). In MA, the migraine attack follows the typical aura consisting of visual, sensory, and/or speech symptoms [2, 3, 7]. Migraine is associated with a number of other conditions and disorders, including neurological conditions, sleep-related disorders, pregnancy-related and gynecologic conditions, obesity, psychiatric conditions, and cardiovascular conditions [3].

Although the pathophysiology of migraine is still poorly understood, the trigeminal-vascular theory proposed 35 years ago is generally accepted [8], in which prostaglandins, especially PGE_2_ and PGI_2_, are considered to play an important role in activating the trigeminal-vascular system (TVS) by involving in the sensitization of peripheral and/or central pain pathways [9, 10]. Cyclooxygenase (COX, also known as prostaglandin-endoperoxide synthase or PTGS) is the key enzyme that catalyzes the formation of prostaglandins from arachidonic acid in response to various stimuli [10]. Two COX isoforms have been identified: COX-1 and COX-2. COX-1 is expressed constitutively with an essentially constant level, but COX-2 is inducible isoform and can be overexpressed in response to pro-inflammatory molecules and can increase prostaglandin production in inflammatory pain [11, 12]. In addition, it was demonstrated, that in rat trigeminal ganglion cells, COX-2 and COX-2-dependent PGE_2_ could induce the development of pain in migraine [13]. In humans, COX-2 expression is higher in patients with migraines than in controls [14], and selective COX-2 inhibitors are equally effective than non-selective COX inhibitors in the treatment of acute migraine [15, 16]. Collectively, COX-2 is considered to be implicated in the pathogenesis of migraine, and there is no difference in COX-2 levels between MA and MO [17].

The human COX-2 gene is located on chromosome 1q25.2-q25.3 and consists of 10 exons spanning 8.3 kb [18]. Several functional single-nucleotide polymorphisms (SNPs) in COX-2 have been widely investigated, including -765 G>C (rs20417) in the promoter region, -1759 G>A (Gly587Arg, rs3218625) in the coding region, and -8473 C>T (rs5275) in the 3’UTR [19–24]. These three SNPs are related to COX-2 expression level and transcription activity [21–24]. Reportedly, the COX-2-8473 C>T polymorphism has an effect on COX-2 mRNA and protein levels [19], the COX-2-765 G>C polymorphism might modify COX-2 mRNA [20], and the COX-2-1759 G>A polymorphism can influence COX-2 activity [24]. In addition, these SNPs have been demonstrated to be associated with several diseases involving COX-2, including bronchial asthma [23], coronary artery disease [25], cerebral infarction [26], and stroke [27].

Nevertheless, little data is available about the relationship between these SNPs and the risk of migraine [14, 28], especially in Chinese Han individuals. Therefore, the aim of the present study was to investigate the potential role of COX-2-765 G>C, -1759 G>A, and -8473 C>T polymorphisms in migraine susceptibility in a Chinese Han population.

## Material and methods

### Study design and subjects

This was a retrospective case-control study of 110 unrelated Han Chinese patients with migraine and 108 healthy controls recruited between March 2014 and August 2016 at the First Affiliated Hospital of Nanjing Medical University and the First People's Hospital of Lianyungang City. The study was approved by the ethics committee of the Affiliated Hospital of Kangda College of Nanjing Medical University/The First People’s Hospital of Lianyungang, Lianyungang. All participants provided written informed consent.

The inclusion criteria were: 1) outpatient at the Neurology department; and 2) diagnosis of migraine determined based on strict neurological examination, computed tomography (CT) or magnetic resonance imaging (MRI), and detailed questionnaire according to the International Classification of Headache Disorders (ICHD-II) criteria [29]. The exclusion criteria were: 1) mental disorders; 2) cardiovascular or cerebrovascular disease, cancer, asthma, anxiety, or depression; or 3) migraine proven to be secondary to another disease.

Non-headache healthy controls were recruited from the physical examination department and were age- (±5 years) and gender-matched with the patients. They were free from any organic diseases or psychiatric disorders and recruited from the same geographic areas. Subjects who received ACE inhibitors were excluded from the study.

### DNA extraction and genotyping

A 2-ml peripheral blood sample was collected from each patient in EDTA-containing tubes. Genomic DNA was extracted using the Blood DNA Extraction Kit ((TiangenBiotect [Beijing] Co., Ltd., Beijing, China). DNA samples were stored at -20ºC until analysis.

Each SNP was analyzed using the ligase detection reaction-polymerase chain reaction (LDR-PCR) sequencing method using an ABI 9600 system (Applied Biosystems, Foster City, CA, USA) [30]. The sequences of the primers are shown in Table 1. The PCR cycling parameters were: 1) 94 ºC for 3 min; 2) 35 cycles of 94ºC for 30 s, 55ºC for 30 s, and 72ºC for 90 s; and 3) final extension at 72ºC for 3 min. For each SNP, two discriminating probes and one common probe were designed and synthesized by Shanghai Generay Biotech Co., Ltd. (Shanghai, China). The common probe was phosphorylated at the 5’ end and labeled at the 3’ end with the fluorophore FAM. The probes of LDR are shown in Table 1. LDR was conducted in a total volume of 10 μl containing 3 μl of PCR product, 1 μl of 10× Taq DNA ligase buffer, 0.125 μl of 40 U/μl Taq DNA ligase, 1 μl of 10 pmol probes (0.01 μl each of probe), and 4.875 μl of ddH_2_O. The ligation reaction was performed with 30 cycles of 94ºC for 30 s and 56ºC for 3 min. Then, 1 μl of the ligation product was mixed with 8 μl of loading dye (marker included) and denatured at 95ºC for 3 min followed by immediate cooling on ice. Ligation samples were analyzed using a 3730XL DNA analyzer (Applied Biosystems, Foster City, CA, USA). In order to validate and confirm the results, 10% of the samples were selected for sequencing to validate the LDR-PCR results [31, 32].

**Table 1:** Primer sequences and the probes for LDR.

SNP	Primer sequences	Probes of LDR
**COX-2 -765G>C**	Forward: 5'-GCC TTA AGG CAT ACG TTT TGG-3'	TC: TTT TTT TTT TTT TTA TGA GGA GAA TTT ACC TTT CCC **C**
	Reverse: 5'-TAC TGT TCT CCG TAC CTT CAC-3'	TG: TTT TTT TTT TTT TTT TTA TGA GGA GAA TTT ACC TTT CCC **G**
		TR: -P-CCT CTC TTT CCA AGA AAC AAG GAG GTT TTT TTT T-FAM-
**COX-2 -8473T>C**	Forward: 5'-CAC TGT CGA TGT TTC CAA TGC-3'	TC: TTG AAA TTT TAA AGT ACT TTT GGT **C**
	Reverse: 5'-ACA GGT GAT TCT ACC CTA TG-3'	TT: TTT TTG AAA TTT TAA AGT ACT TTT GGT **T**
		TR: -P-ATT TTT CTG TCA TCA AAC AAA AAC AGG T-FAM-
**COX-2 -1759G>A**	Forward: 5'-TCA TTC AGT GTT CCA GAT CC-3'	TA: TTT TAT CAA TGC AAG TTC TTC CCG CTC C**A**
	Reverse: 5'-CGC AAC AGG AGT ACT GAC TT-3'	TG: TTT TTT TAT CAA TGC AAG TTC TTC CCG CTC C**G**
		TR: -P-GAC TAG ATG ATA TCA ATC CCA CAG TTT TTT TT-FAM-

### Statistical analysis

The continuous data were presented as means ± standard deviation and were analyzed using the Student t-test. The categorical data were presented as numbers and percentages and analyzed using the chi-square test. The distributions of the genotypes and alleles between patients with migraine and healthy controls were compared using the chi-square test. The odds ratios (ORs) with 95% confidence intervals (95% CIs) were calculated. The goodness-of-fit chi-square test was used to evaluate the Hardy-Weinberg equilibrium (HWE) in the control subjects. Statistical analyses were performed using SPSS 18.0 (IBM, Armonk, NY, USA). Two-sided P-values <0.05 were considered statistically significant.

## Results

### Characteristics of the subjects

The mean age of the 110 patients (73 females, 37 males) was 32.7±9.7 years (Table 2). The mean age of the 108 controls (72 females, 36 males) was 33.9±9.1 years. There were no significant differences between the two groups in terms of age and sex (P = 0.349 and P = 0.962, respectively). The migraine cohort included 55 (50.0%) patients with a family history of migraines and 55 (50.0%) without. One (0.9%) patient had hypertension, 14 (12.7%) had a smoking history, and 18 (16.4%) had a drinking history. The course of the disease was 10 (range, 6–16.5) years, the frequency of onset was 2 (range, 1.5–3.5) per month, and the duration of attacks was 24 (range, 12–27) h.

**Table 2:** Characteristics of the patients.

Variable	Migraine	Controls	P
	n = 110	n = 108	
Sex			0.962
Men	37 (33.6%)	36 (33.3%)	
Women	73 (66.4%)	72 (66.7%)	
Age	32.7±9.7	33.9±9.2	
Median (IQR)	30 (25, 40)	33 (26, 41.5)	0.368
Aura	16 (14.5%)	-	
Course of disease (years)	10 (6, 16.5)	-	
Frequency of onset (time/month)	2 (1.5, 3.5)	-	
Duration of migraine attacks (h)	24 (12, 27)	-	
Family history	55 (50%)		
Hypertension	1 (0.9%)		
Smoking history	14 (12.7%)	-	
Drinking history	18 (16.4%)		

### COX-2 genotypes

The polymorphisms were detected by LDR-PCR analysis (S1 Checklist). For confirmation of the SNPs found by LDR-PCR, 10% of the samples were randomly selected for sequencing, and the consistency rate was 100% (S1 File). The genotype and allele distributions of the COX-2-765G>C, -1759G>A, and -8473T>C polymorphisms are shown in Tables 3 and 4. The genotypes of these three SNPs in controls followed the Hardy-Weinberg equilibrium (P = 0.215, P = 0.884, and P = 0.689, respectively). The frequency of the genotypes and alleles of the COX-2-1759G>A polymorphism were significantly different between the migraine and control groups (P = 0.038 and P = 0.040, respectively). Compared to the COX-2-1759AG genotype carriers, the GG genotype carriers had a higher disease risk (OR = 8.720, 95%CI: 1.072–70.960, P = 0.038) for migraine. The frequency of the COX-2-1759A allele in controls was significantly higher compared with that in the migraine group (OR = 0.119, 95%CI: 0.015–0.957, P = 0.040). The COX-2-1759A allele indicated a decreased risk for the development of migraine compared with that of the 1759G allele. There were no differences in the genotype distribution of COX-2-8473T>C (P = 0.346) and COX-2-765G>C (P = 0.330) polymorphisms between the two groups (Table 3).

**Table 3:** Genotype distributions of the COX-2-765G>C, -8473T>C, and -1759G>A polymorphisms.

SNP	Control (n = 108) (%)	Migraine (n = 110) (%)	P	MA^1^ (n = 16) (%)	P	MO^2^ (n = 94) (%)	P	F-migraine^3^ (n = 73) (%)	P	M-migraine^4^ (n = 37) (%)	P
**COX-2-765G>C**			0.346		>0.999		0.230		0.348		0.815
GG	105 (97.2)	103 (93.6)		16 (100.0)		87 (92.6)		68 (93.2)		35 (94.6)	
CG	3 (2.8)	7 (6.4)		0		7 (7.4)		5 (6.8)		2 (5.4)	
CC	0	0		0		0		0		0	
CC+ CG/GG	3/105	7/103	0.346	0/16	>0.999	7/87	0.230	73/0	0.348	37/0	0.815
GG+ CG/CC	108/0	110/0	**-**	16/0	**-**	94/0	**-**	5/68	**-**	2/35	**-**
**COX-2-8473T>C**			0.33		0.879		0.485		0.867		0.069
TT	76 (70.4)	74 (67.3)		11 (68.8)		63 (67.0)		53 (72.6)		21 (56.8)	
CT	27 (25.0)	34 (30.9)		5 (31.2)		29 (30.9)		18 (24.7)		16 (43.2)	
CC	5 (4.6)	2 (1.8)		0		2 (2.1)		2 (2.7)		0	
CC+ CT/TT	32/76	36/74	0.622	5/11	>0.999	31/63	0.608	20/53	0.745	16/21	0.129
TT+ CT/CC	103/5	108/2	0.428	16/0	>0.999	33/63	0.559	71/2	0.800	37/0	0.418
**COX-2-1759G>A**			0.038^*^		0.562		0.071		0.138		0.198
GG	100 (92.6)	109 (99.1)		16 (100.0)		93 (98.9)		72 (98.6)		37 (100)	
AG	8 (7.4)	1 (0.9)		0		1 (1.1)		1 (1.4)		0	
AA	0	0		0		0		0		0	
AA+ AG/GG	8/100	1/109	0.038^*^	0/16	0.562	1/93	0.066	73/0	**-**	37/0	**-**
GG+ AG/AA	108/0	110/0	**-**	16/0	**-**	94/0	**-**	1/72	0.138	0/37	0.198

^**1**^MA: migraine with aura.

^**2**^MO: migraine without aura.

^**3**^F-Migraine: female migraine cases.

^**4**^M-Migraine: male migraine cases.

*Significant difference in genotype distribution comparing migraine cases with controls: OR = 8.720, 95%CI = 1.072–70.960.

**Table 4:** Allele frequencies of the COX-2-765G>C, -8473T>C, and -1759G>A polymorphisms.

SNP	Control (n = 108) (%)	Migraine (n = 110) (%)	P	MA^1^ (n = 16) (%)	P	MO^2^ (n = 94) (%)	P	F-Migraine^3^ (n = 73) (%)	P	M-Migraine^4^ (n = 37) (%)	P
**COX-2-765G>C**											
G	213 (98.6)	213 (96.8)	0.352	32 (100.0)	>0.999	181 (96.3)	0.236	141 (96.6)	0.353	72 (97.3)	0.817
C	3 (1.4)	7 (3.2)		0		7 (3.7)		5 (3.4)		2 (2.7)	
**COX-2-8473T>C**											
T	179 (82.9)	182 (82.7)	0.968	27 (84.4)	0.832	155 (82.5)	0.911	124 (84.9)	0.602	58 (78.4)	0.388
C	37 (17.1)	38 (17.3)		5 (15.6)		33 (17.5)		22 (15.1)		16 (21.6)	
**COX-2-1759G>A**											
G	208 (96.3)	219 (99.6)	**0.040**^#^	32 (100.0)	0.568	187 (99.5)	0.069	145 (99.3)	0.143	74 (100)	0.205
A	8 (3.7)	1 (0.4)		0		1 (0.5)		1 (0.7)		0	

^**1**^MA: migraine with aura.

^**2**^MO: migraine without aura.

^**3**^F-Migraine: female migraine cases.

^**4**^M-Migraine: male migraine cases.

^**#**^Migraine cases compared with controls by allele: OR = 0.119, 95%CI = 0.015–0.957.

### Subgroup analyses

Subgroup analyses were performed regarding MA vs. MO and regarding migraine in females vs. males. The three polymorphisms were not associated with the type of migraine or gender (all P>0.05) (Tables 3 and 4).

### Multivariable analyses

Table 5 presents the multivariable analyses. After adjustment for age and sex, the COX-2-1759A was associated with migraine (OR = 0.118, 95% CI 0.014 to 0.962, P = 0.046).

**Table 5:** Univariable and multivariable analyses.

Variables	Univariable			Multivariable*		
	OR	OR 95% CI	P-value	OR	OR 95% CI	P-value
COX-2-765G>C						
Dominant model (CG+CC vs. GG)	2.379	(0.599, 9.451)	0.218	2.387	(0.599, 9.516)	0.217
COX-2-8473T>C						
TT	1					
CT	1.293	(0.711, 2.352)	0.399	1.274	(0.696, 2.334)	0.432
CC	0.411	(0.077, 2.184)	0.297	0.408	(0.076, 2.184)	0.295
Dominant model (CT+CC vs. TT)	1.155	(0.651, 2.051)	0.622	1.14	(0.638, 2.037)	0.659
Recessive model (CT+TT vs. CC)	2.621	(0.497, 13.813)	0.256	2.633	(0.497, 13.946)	0.255
COX-2-1759G>A						
Dominant model (AG+AA vs. GG)	0.115	(0.014, 0.933)	0.043	0.118	(0.014, 0.962)	0.046

*: Adjusted age and sex.

## Discussion

COX-2 is known to be involved in the pathogenesis of migraine [13–16], and some polymorphisms are known to affect the expression of COX-2 [14, 20, 24, 28]. Therefore, this study aimed to explore the associations between the -765 G>C (rs20417), -1759 G>A (rs3218625), and -8473 C>T (rs5275) COX-2 polymorphisms and migraine in Chinese Han individuals. The COX-2 1759A allele might be involved in the development of migraine in Chinese Han individuals. This is the first study, to our knowledge, to reveal the effect of 1759G>A polymorphism on migraine. The COX-2 -765G>C and -8473T>C polymorphisms did not have a significant impact on migraines. Nevertheless, large-scale studies are necessary to confirm the results.

The SNP 1759G>A is located at codon 587 of exon 10. This variant leads to a substitution from glycine to arginine in the COX-2 protein, and, in vitro, the 1759A allele has a significantly higher enzymatic activity towards arachidonic acid than the 1759G allele [24]. Therefore, individuals carrying the 1759A allele might have higher activity of this enzyme and higher production of prostaglandins, which are related to migraine [11, 12]. On the other hand, in the present study, the 1759AG heterozygotes were overrepresented in controls compared with patients with migraine, suggesting that the 1759A allele might play a protective role in migraine and that the 1759AG genotype is associated with a reduced risk of migraine. These conflicting results with those of the previous functional study might be partly explained by the fact that the enzymatic activities of COX-2-587Gly and COX-2-587Arg were measured in vitro and that the sample size was small (n = 18) in the functional study.

On the other hand, the functional effects of the COX-2-765G>C and -8473T>C SNPs have been investigated. The -765G>C polymorphism is in the promoter region of COX-2 and seems to have a functional impact on transcription [22]. The COX-2-8473T>C polymorphism was reported to be related to alterations at the COX-2 mRNA and protein levels as sequences within the 3’UTR are important for mRNA stability and translational efficiency [19, 33]. Previous studies suggested that the -765C allele had a lower promoter activity and significantly reduced COX-2 expression compared with the -765G allele [34], and the individuals with 8473 CC genotype showed significantly higher plasma COX-2-dependent PGE_2_ levels [35]. Dasdemir et al. [28] reported that the COX-2-765C allele was related to a decreased risk of migraine in individuals from Turkey. In Iranians, the COX-2-765CC, -765CG, -1195GG, and -1195AG genotypes were associated with an increased risk of migraine [36]. On the other hand, a recent in colorectal cancer indicated that the COX-2-765G>C and -8473T>C polymorphisms did not alter the PTGS-2 mRNA levels and COX-2 activity [37]. In the present case-control study, there were no associations between migraine and the COX-2-765G>C and -8473T>C polymorphisms in Chinese Han individuals. These conflicting results may suggest that the effect of the COX-2 polymorphisms on mRNA levels and disease risks could be influenced by the ethnic background or the environment. Further investigations are needed to confirm these results.

It has been shown that the COX-2-765G>C polymorphism is a protective factor against ischemic stroke in Italians [34], but the association between COX-2-765G>C and ischemic stroke and leukoaraiosis could not be replicated in Chinese [27, 38] and Koreans [39]. As for COX-2-8473T>C, Maguire et al. [40] demonstrated a significant effect of the COX-2-8473T>C polymorphisms on ischemic stroke functional outcome in Australians, while no association between the COX-2-8473T>C polymorphisms and intracerebral hemorrhage was observed in Koreans [41]. Similarly, a previous study reported that COX-2-765G>C was associated with migraine in Turkish individuals [28]. Taken together, those results from different countries suggest genetic and phenotypic heterogeneity among the diverse ethnic populations.

A number of limitations of the present study should be considered. First, the sample size may not be large enough to allow a definitive conclusion. Second, migraine is a complex and multifactorial disorder [42, 43], and the migraine phenotype is probably influenced by many genetic variants and environmental factors, which means that each genetic variant only confers a small to moderate change in the risk of migraine [44]. Third, selection bias may occur because the patients with migraines and the controls in this study were from one province in China. Nevertheless, the genotypes of the three SNPs in controls followed the Hardy-Weinberg equilibrium. Fourth, this is the first time 1749G>A is investigated in Chinese Han individuals, lacking evidence from other ethnic populations. Fifth, the pressure at spiritual and physical levels can affect the occurrence of migraines, but they were not assessed in this study. Sixth, age was taken into account, but not in terms of pressures from spiritual and physical levels in both groups. Seventh, there were too few factors related to migraine in the multivariate analysis. Finally, in view of the case-control study only investigated in the Chinese Han population, it needs caution when considering other ethnic populations.

In conclusion, the COX-2-1759A allele might be involved in migraine development in Chinese Han individuals. Further functional investigations and independent cohorts are needed to clarify the effect of the COX-2 gene polymorphisms on migraine susceptibility, but this will have to be confirmed in large-scale studies and across multiple ethnicities and populations.

## Supporting information

S1 Checklist STROBE statement. (DOC)

S1 File Typical sequencing data. (DOCX)
