# Supplementary material for: Association of the cyclooxygenase-2 1759A allele with migraine in Chinese Han individuals
Source: PLoS One. 2020 Sep 30;15(9):e0239856. doi: 10.1371/journal.pone.0239856 (PMC7526883; doi:10.1371/journal.pone.0239856)

**S1 File.** Typical sequencing data.

**S1: rs5275**

TTGAAATTTTAAAGTACTTTTGGT[C/T]ATTTTTCTGTCATCAAACAAAAA


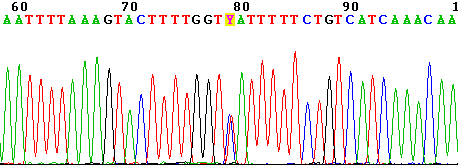


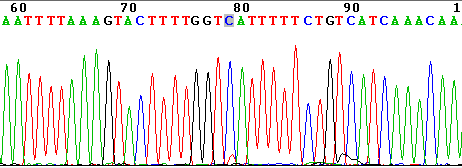


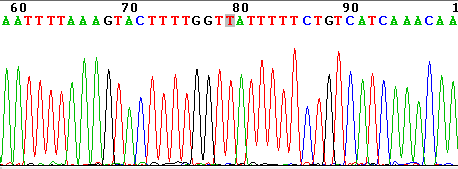


**S2: rs20417**

TATGAGGAGAATTTACCTTTCCC[C/G]CCTCTCTTTCCAAGAAACAAGGA


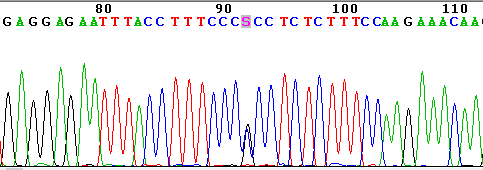


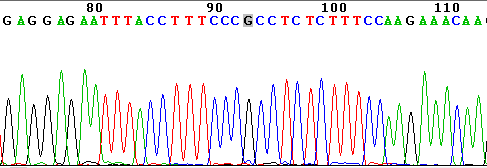


**S3: rs3218625**

TCAATGCAAGTTCTTCCCGCTCC[A/G]GACTAGATGATATCAATCCCACAG


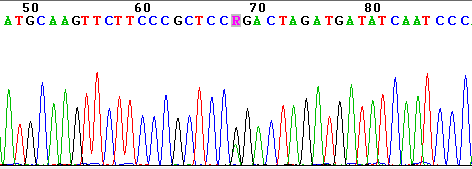


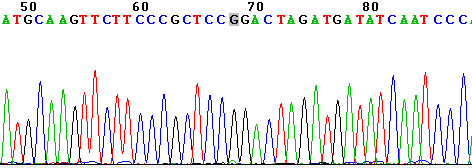

Supplement: S1 File — (DOCX) [file pone.0239856.s002.docx]
